# Ifit2 deficiency restricts microglial activation and leukocyte migration following murine coronavirus (m-CoV) CNS infection

**DOI:** 10.1371/journal.ppat.1009034

**Published:** 2020-11-30

**Authors:** Jayasri Das Sarma, Amy Burrows, Patricia Rayman, Mi-Hyun Hwang, Soumya Kundu, Nikhil Sharma, Cornelia Bergmann, Ganes C. Sen

**Affiliations:** 1 Department of Inflammation and Immunity, Lerner Research Institute, Cleveland Clinic, Ohio, United States of America; 2 Department of Biological Sciences, Indian Institute of Science Education and Research Kolkata, Mohanpur, West Bengal, India; 3 Department of Neurosciences, Cleveland Clinic, Ohio, United States of America; Thomas Jefferson University, UNITED STATES

## Abstract

The interferon-induced tetratricopeptide repeat protein (Ifit2) protects mice from lethal neurotropic viruses. Neurotropic coronavirus MHV-RSA59 infection of Ifit2^-/-^ mice caused pronounced morbidity and mortality accompanied by rampant virus replication and spread throughout the brain. In spite of the higher virus load, induction of many cytokines and chemokines in the brains of infected Ifit2^-/-^ mice were similar to that in wild-type mice. In contrast, infected Ifit2^-/-^ mice revealed significantly impaired microglial activation as well as reduced recruitment of NK1.1 T cells and CD4 T cells to the brain, possibly contributing to the lack of viral clearance. These two deficiencies were associated with a lower level of microglial expression of CX3CR1, the receptor of the CX3CL1 (Fractalkine) chemokine, which plays a critical role in both microglial activation and leukocyte recruitment. The above results uncovered a new potential role of an interferon-induced protein in immune protection.

## Author summary

Interferons (IFNs) are known to protect from virus dissemination and pathogenesis. Several IFN stimulated genes (ISG) regulate neuropathogenesis but the mechanisms underlying the antiviral effects are not clearly understood. IFN induced tetratricopeptide repeats (Ifit) are a class of ISGs. Among the Ifits, Ifit2 is known to play a beneficial role in restricting neurotropic viral replication. To provide better cellular insights into the protective mechanisms of Ifit2 functions, using a neurotropic coronavirus infection in Ifit2 depleted mice we report that in the absence of Ifit2, viral replication is dramatically increased and mice develop severe clinical signs and symptoms of neurological deficit. Despite the enormous viral load, Ifit2 deficient mice are impaired in microglial activation and recruitment of peripheral leukocytes into the CNS. This impaired leuocyte infiltration in Ifit2 deficient mice was also associated with reduced expression of a novel chemokine receptor CX3CR1,which is important for viral induced microglial activation and maintaining tissue homeostasis.

**Highlights**

1. Ifit2 protects mice from uncontrolled coronavirus replication in the brain.
2. Microglial activation in the CNS is impaired in infected Ifit2^-/-^ mice.
3. Consequently, peripheral lymphocyte migration to the CNS is restricted.
4. Impaired microglial activation is associated with decreased CX3CR1 expression.

## Introduction

Viral infection in the CNS initiates activation of cellular sensors like Toll like receptor (TLRs)/ Rig-I- like receptor (RLRs)/ synthase for the second messenger cyclic GMP–AMP and the cyclic GMP–AMP receptor stimulator of interferon genes (cGAS-STING) [1–3], which in turn activate transcription factors such as Interferon regulatory factors (IRFs), Nuclear Factor kappa-light-chain-enhancer of activated B cells (NF-κB) and downstream type I interferon (IFN) genes. Secreted IFNβ and members of the IFNα family bind to Interferon-α/β receptor (IFNAR), which induces the expression of more than 200 IFN stimulated genes (ISG)[4–7]. Among these ISGs are the *i*nter*f*eron-*i*nduced proteins with *t*etratricopeptide repeats (IFITs)[2,8,9]. There are four IFIT members in humans and three Ifit members in mice [7,10]. Ifit proteins are known to inhibit virus replication by binding and regulating the functions of cellular and viral proteins and RNAs [1]. The recent use of genetically engineered Ifit knockout mouse models have revealed that amongst these Ifit proteins, Ifit2 mostly restricts viral infection and protects mice from severe pathogenesis and mortality following Rabies Virus [11], lethal Vesicular Stomatitis Virus (VSV) [12,13], West Nile Virus (WNV) [14], and Sendai virus (SeV) infection [15]. Similarly, adult 6–7 week old Ifit2^-/-^ mice intracranially infected with a dual hepatotropic and neurotropic strain of mouse hepatitis virus (vMHV-GFP), a coronavirus, exhibited pronounced disease severity and a mortality rate up to 60% compared to benign disease in wildtype (WT) mice. Elevated infectious viral load in the brains of Ifit2^-/-^ mice coincided with impaired IFNα/β production and ISG upregulation with no deficits in IFNℽ production [16]. vMHV-GFP was derived from a MHV-A59 cDNA clone (vMHV-inf-1) in which the accessory gene 4 was replaced by a gene encoding a fusion protein of EGFP and a lymphocytic choriomeningitis virus-derived cytotoxic T lymphocyte epitope described previously [17].

While the vMHV-GFP studies in Ifit2^-/-^ mice demonstrated impaired IFNα/β mRNA upregulation, additional roles of Ifit2 in regulating microglial activation were not explored. As microglia/macrophages have been reported to contribute in significant ways to IFNα/β secretion in the inflamed brain [18], the absence of Ifit2 may also affect microglia-neuronal interactions and leukocyte localization in establishing innate immune responses. Current literature in different CNS diseases showed that strong neuronal excitation in the inflamed brain is communicated through C-X3-C motif chemokine ligands, i.e. CX3CL1 chemokine (fractalkine) [19,20]. CX3CR1 and CX3CL1 is ubiquitously expressed throughout the organism but their specific expression in any given tissue is highly cell-specific, which is clearly evident in the CNS, where CX3CR1 expression is only observed in microglia and CX3CL1 expression is found on neurons [21–23]. During neuronal excitation, the soluble form of this fractalkine is cleaved from the neuronal surface and interacts with its cognate receptor, CX3CR1, predominantly expressed on microglia. Studies also revealed that CX3CR1 expression on microglia plays an important role in synaptic pruning during normal brain development, establishing its function in CNS homeostasis [24]. This interaction establishes neuron-microglial communication and may signal for subsequent microglial activation. Overall, CX3CR1 is thought to function as a dampening regulator to limit microglial detrimental functions during homeostasis [25]. Following microglial activation in the inflamed brain, CX3CL1 is induced in endothelial cells [26,27]. Thus, the CX3CR1-CX3CL1 axis also serves as a leukocyte trafficking regulator providing hybrid functions of adhesion as well as migration [20,28,29].

To look into additional regulatory roles of Ifit2 in the context of innate immune activation, we used a isogenic spike protein recombinant EGFP expressing MHV-A59 strain designated as RSA59, known to be less lethal, but induce acute neuroinflammation and chronic demyelination and axonal loss in 4-5-week-old mice. Infection with 20,000 PFUs RSA59 elicits similar disease as 4,000 PFUs of the parental WT MHV-A59 strain [30,31]. Bright EGFP expression in the brains of RSA59 infected mice greatly facilitates analysis of viral infection and spread. Furthermore, the pathogenesis of intracranial RSA59 infection of 4 week old C57BL/6 mice is well described [17,30–36]. The virus infects CNS resident cells and activates astrocytes and microglia to produce chemokines and cytokines, which facilitate leukocyte recruitment and migration into the CNS parenchyma through the blood brain barrier (BBB). Activated circulating leukocytes and monocytes start to accumulate in the CNS blood vessels, resulting in perivascular cuff formation [32,34]. CNS resident microglia and migratory monocytes further start to form a clustered nodule surrounding infected neuronal cells, also termed as ‘microglial nodule’ formation, which constitutes a signature of CNS viral infection and encephalitis [33,37]. At day 3–5 p.i., virus spreads from the brain to the brain stem region and to the gray matter of the spinal cord. By day 5–7 p.i., virus spreads from the gray matter to the white matter through axonal transport [32,33]. Acute myelitis (day 5–7 p.i.) progresses to chronic neuroinflammatory demyelination with consecutive axonal loss which reaches its peak at day30 p.i. [34].

Similar to previous findings using vMHV-GFP [16], our results show that Ifit2^-/-^ mice develop severe neurological symptoms during day 4–7 p.i. resulting in 70% mortality by day 7–8 p.i. at 2,000 PFUs (one tenth of the RSA59 LD_50_ dose). Additionally, viral load and viral spread were significantly increased in the brains in the absence of Ifit2. Interestingly, despite increased viral load with minimal changes in the expression of selected cytokines and chemokines, Ifit2^-/-^ mice exhibited reduced encephalitis as evident by decreased perivascular cuffing, and reduced formation of microglial nodules. Flow cytometry data confirmed that migration of CD45^hi^ peripheral leukocytes was significantly lower in Ifit2^-/-^ infected mice compared to wildtype (WT) infected mice. While no significant changes were observed in the number of CD45^hi^ CD11b^+^ (neutrophil and monocyte derived macrophages), there was a significant reduction in the accumulation of lymphocytes (NK-1.1^+^ T, CD4^+^ T cells) in Ifit2^-/-^ relative to WT mice. CD45^lo/int^ CD11b^+^ cells showed no changes in the absolute number between both groups. Despite no evidence of alterations in myeloid cell numbers, morphometric histopathological analysis revealed significantly reduced microglia/macrophage activation in the absence of Ifit2. Surprisingly, microglial CX3CR1 expression was significantly lower in Ifit2^-/-^ compared to WT mice following infection, although levels were similar in naïve mice. The reduction in CX3CR1 expression could be a possible restricting factor resulting in impaired microglial function and lymphocyte migration and thus compromised host immunity in Ifit2^-/-^ infected mice.

## Results

### Ifit2 deficiency increased disease severity and viral load

**Fig 1:**
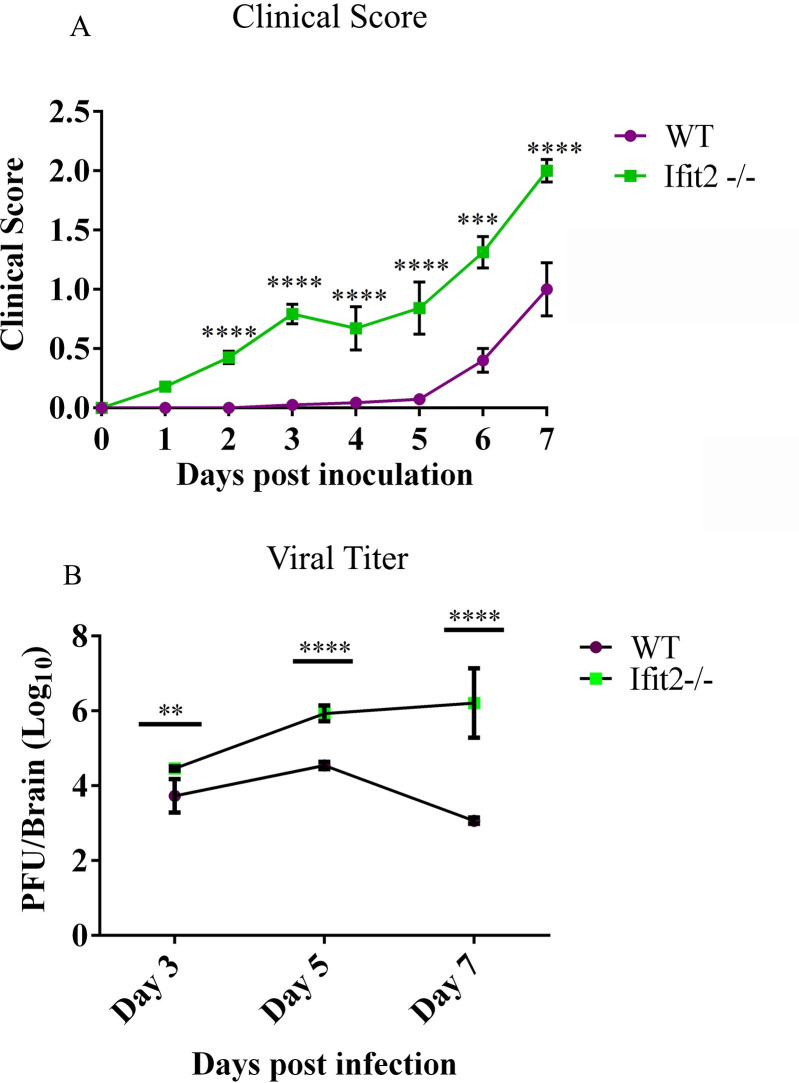
Ifit2^-/-^ mice showed increased disease severity and increased infectious viral load upon RSA59 infection. : 4–5 weeks old WT (n = 28) and Ifit2^-/-^ mice (n = 30) were infected intracranially with 2000 PFU of RSA59 and monitored for development of clinical disease (A) and viral titers in brains (B). Clinical scores were assigned by an arbitrary scale of 0–4 as described in Materials and Methods. Viral burden in brain tissue homogenates was analysed by plaque assay and shown as PFU/ brain (approx. 300 mg) from individual mice (n = 5-6/timepoint). The solid line represents the mean viral titer. Asterix (*) indicate statistical significance by Two-Way ANOVA analysis for clinical score and unpaired t-test for viral titers. (**P<0.01, ***P<0.001, ****P<0.0001).

Previous studies revealed a critical role of Ifit2 in protecting adult mice from vMHV-GFP induced encephalitis and mortality by limiting viral spread and enhancing IFNα/β responses [16].To determine if Ifit2 played a similar antiviral role against RSA59 infection in younger 4-5-week-old mice, infected WT and Ifit2^-/-^ mice were monitored for development of clinical disease. The majority of the infected WT mice displayed mild disease scores of 0.5–1, indicated by ruffled fur and occasionally slightly hunched back, starting at day 5 p.i., with 100% survival (Fig 1A). In contrast, clinical disease in Ifit2^-/-^ mice (0.5) was evident as early as day 2 p.i., with disease severity increasing to a score of 2–2.5 by day 7 p.i., after which almost 70% of mice became moribund (clinical score = 3.5) or died (clinical score = 4). The few surviving Ifit2^-/-^ mice showed higher disease severity and slower recovery rate than WT mice. These data confirmed that Ifit2 plays a protective role against RSA59 infection (Fig 1A).

To confirm whether the increased disease severity correlated with uncontrolled viral replication, viral titers in brains of infected WT and Ifit2^-/-^ mice were assessed by plaque assay. Infectious virus was higher in Ifit2^-/-^ compared to WT mice at day 3 p.i. and differences in infectious viral load significantly increased by day 5 p.i. While WT mice started to control viral replication by day 7 p.i., Ifit2^-/-^ mice showed no signs of virus control, as evident by increased infectious virus (Fig 1B).

### Ifit2 deficiency increased viral spread throughout the brain parenchyma

**Fig 2:**
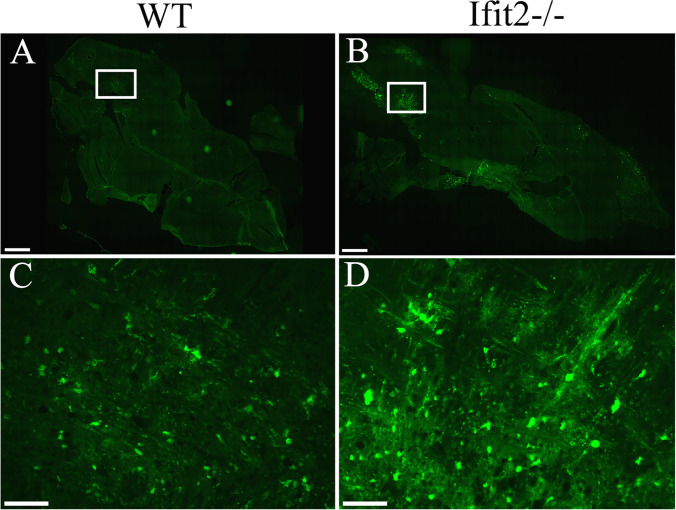
Ifit2 deficiency increases RSA59 viral spread throughout the brain parenchyma. 5–10 μm thin cryosections were prepared from brains of 4–5 weeks old WT and Ifit2^-/-^ infected mice at day 5 p.i. Representative EGFP fluorescence in whole brain section is shown from WT (A; n = 2) and Ifit2^-/-^ (B; n = 4) mice. Similar anatomic locations from the WT and Ifit2-/- mice brain stem regions were magnified for both WT (C) and Ifit2^-/-^ (D) infected mice. Scale bar for Panels A and B = 1mm and for Panel C and D = 50 μm.

RSA59 infects different regions of the brain including olfactory bulb, cortex, basal forebrain, hippocampal region, brainstem, and deep cerebellar white matter in 4-5-weeks-old WT mice [38]. To determine whether Ifit2 preferentially protects distinct brain regions, cryosections from brain tissues were scanned for EGFP expression to detect virus infected cells. Although no differences were observed in the overall distribution of RSA59 infected cells between WT (Fig 2A) and Ifit2^-/-^ mice (Fig 2B), expression of total EGFP was more robust and quantifiable in all anatomical regions in Ifit2^-/-^ mice (Fig 2D) compared to WT mice (Fig 2C). The boxed region represents brainstem (Fig 2). The increased expression of EGFP supports the correlation between increased viral load, accelerated disease onset and more severe disease symptoms following RSA59 infection in Ifit2^-/-^ compared to WT mice.

### Ifit2 deficiency decreased viral induced cellular inflammation and microglia/macrophage activation

**Fig 3:**
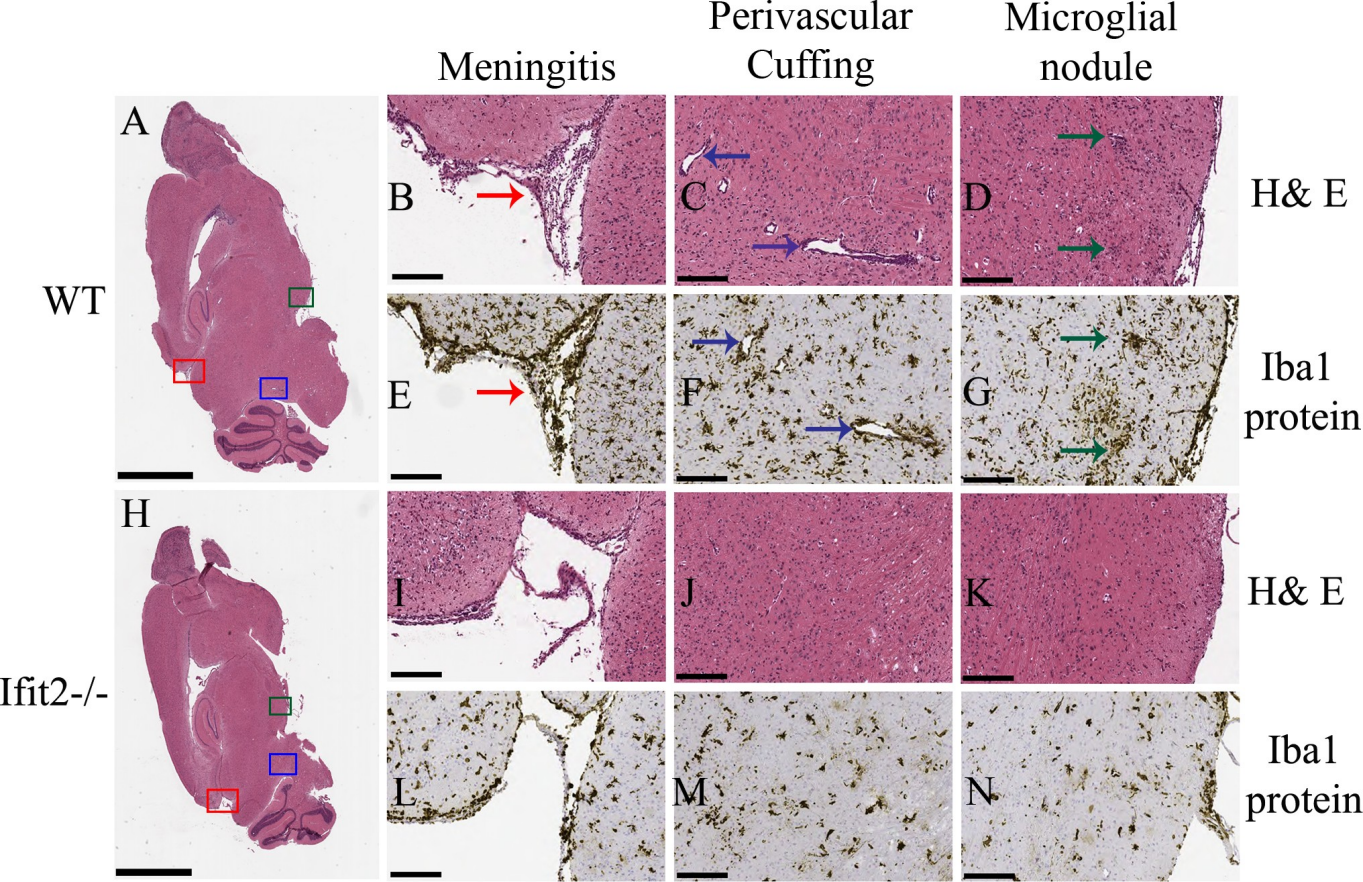
Ifit2 deficiency results in decreased neuroinflammation upon RSA59 infection. 5 μm thin paraffin embedded serial sections of RSA59 infected WT and Ifit2^-/-^ brain tissues processed for H & E (Panels A-D and H-K) and Iba1 staining (Panels E-G and L-N) at 5 days p.i. as indicated. Representative scanned images of whole brain are shown for WT (A) and Ifit2^-/-^ (H) mice with selected meningeal infiltration indicated by Red box, perivascular cuffing by blue box and microglial nodule formation by green box. Selected boxed areas are enlarged for WT and Ifit2^-/-^ brains in Panels B, C, D and Panels I, J, K, respectively. Representative Iba1 staining in meninges, perivascular cuffs and microglial nodules is shown for WT and IFIT2^-/-^ brains in Panels E, F,G and L,M,N, respectively. Ifit2^-/-^ mice showed reduced overall inflammation, reduced or no perivascular cuffing and only scattered Iba-1+ cells without apparent nodule formation. Red arrow depicts menegitis, Dark blue arrow depicts encephalitis (perivascular cuffing), green arrow shows encephalitis (microglial nodules). The experiment was repeated three times and total n = 8. The scale bar for Panel A and H is 2 mm and for Panel B-G and Panel I-N is 75 μm.

To assess, if the increased viral load was associated with increased cellular inflammation, we assessed the histopathological changes by Hematoxylin / Eosin (H&E) staining and microglial activation by Iba1 staining at day 5 p.i., at the peak of virus replication. H&E staining for whole brain is shown for WT mice (Fig 3A) and Ifit2^-/-^ mice (Fig 3H). WT mice exhibited meningeal infiltrates (Fig 3B, red arrow), perivascular cuffing (blue arrow in Fig 3C) and microglial nodules (indicated by green arrow Fig 3D). Staining for Iba1 reactivity to mark activated microglia and macrophages, revealed similar staining patterns as H&E reactivity (Fig 3E–3G). Formation of microglial nodules proximal to perivascular sites and in the parenchyma was also evident (Fig 3F and 3G). By contrast, Ifit2^-/-^ mice showed very mild meningeal and CNS parenchymal infiltration (Fig 3I) and little evidence for perivascular cuffing (Fig 3J) or microglial nodule formation (Fig 3K) by H&E staining at similar anatomical regions as those of WT infected mice. Iba-1 reactivity was also significantly reduced (Fig 3L–3N) in the meninges (Fig 3L) and no significant microglial nodule formation was evident in the brain parenchyma (Fig 3M and 3N). These data indicated that inflammatory responses and microglia/macrophage activation were impaired in the absence of Ifit2.

**Fig 4:**
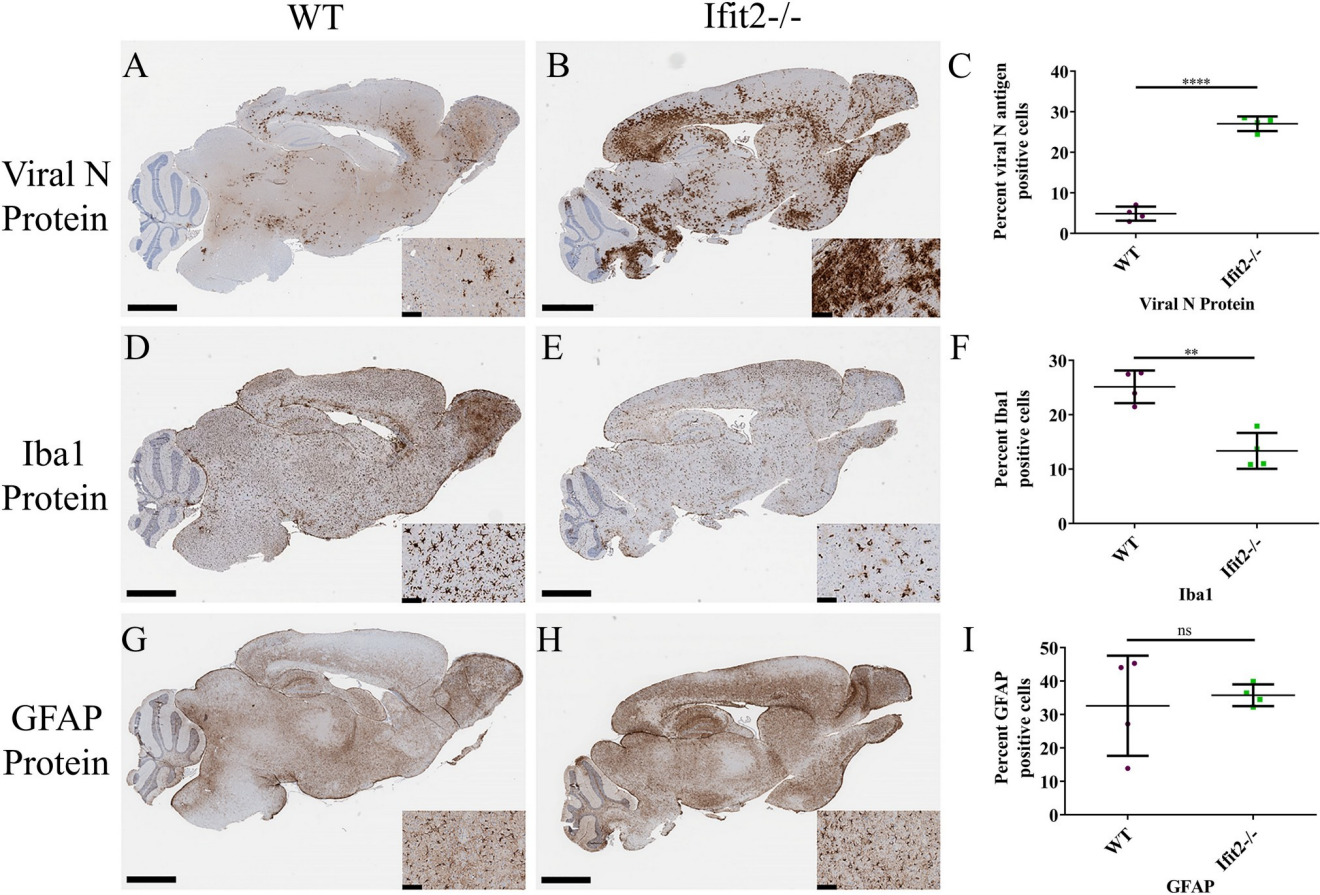
Ifit2 deficiency results in increased viral infection, impaired microglial activation but induce no changes in astrocyte activation upon RSA59 infection. 5 μm thin serial sections from brain tissues used in Fig 3 were processed for Viral N protein (Panels A,B), Iba1 (Panels D,E) and GFAP (Panels G,H) immunostaining as indicated. Quantification of Viral N protein, Iba1 and GFAP expression are graphically represented in Panel C, F, and I respectively. The experiment was repeated three times and total n = 8.The scale bar for the whole brain section is 2.8 mm and for the insets is 80 μm. Asterix (*) represents differences that are statistically significant by Student’s unpaired t-test analysis. (**P<0.01, ****P<0.0001).

Reduced cellular inflammation, despite increased viral load in RSA59 infected Ifit2^-/-^ mice was an unanticipated result. To assess whether this phenomenon was region specific or a global impairment, we assessed the activation pattern of microglia and astrocytes relative to viral antigen positive areas in serial paraffin embedded brain sections from both WT and Ifit2^-/-^ mice (Fig 4). Viral nucleocapsid antigen, detected using anti-nucleocapsid antibody, was present in olfactory bulb, cortex, basal forebrain, hippocampal region, brain stem, and deep cerebellar white matter in both WT (Fig 4A) and Ifit2^-/-^ mice (Fig 4B). However, the staining was significantly more robust in Ifit2^-/-^ mice; quantitative assessment revealed ~5% infected area in WT brains relative to ~28% in Ifit2^-/-^ brains (Fig 4C). Microglial activation detected by Iba1 staining, largely reflected the pattern of virus infection in WT mice (Fig 4D). By contrast, Iba1 staining was significantly reduced in Ifit2^-/-^ brains, even in heavily infected areas (Fig 4E). Overall, Iba1 staining was significantly reduced from 25% to 12% (Fig 4F). Reduced Iba1 reactivity corresponded to reduced microglial morphometric activation in Fig 3. Notably, however, astrocyte activation assessed via GFAP reactivity was similar in both WT and Ifit2^-/-^ mice (Fig 4G–4I). Histopathological studies in combination with immunohistochemistry thus revealed that Ifit2 deficiency is associated specifically with impaired microglia, rather than astrocyte activation, despite the vastly increased viral load.

### Ifit2 deficiency does not affect the expression of selected proinflammatory cytokines and chemokines in the infected brains at mRNA levels

Neurotropic MHV infection in WT mice induces a variety of chemokines and cytokines, which not only promote innate immune responses in resident cells, but also regulate BBB integrity and recruitment of leukocytes with antiviral functions. Chemokine-chemokine receptor interactions transduce signals that results in cytoskeletal reorganization, integrin activation, and other functions leading to increased adhesion and migration of circulating leukocytes to the CNS.

**Fig 5:**
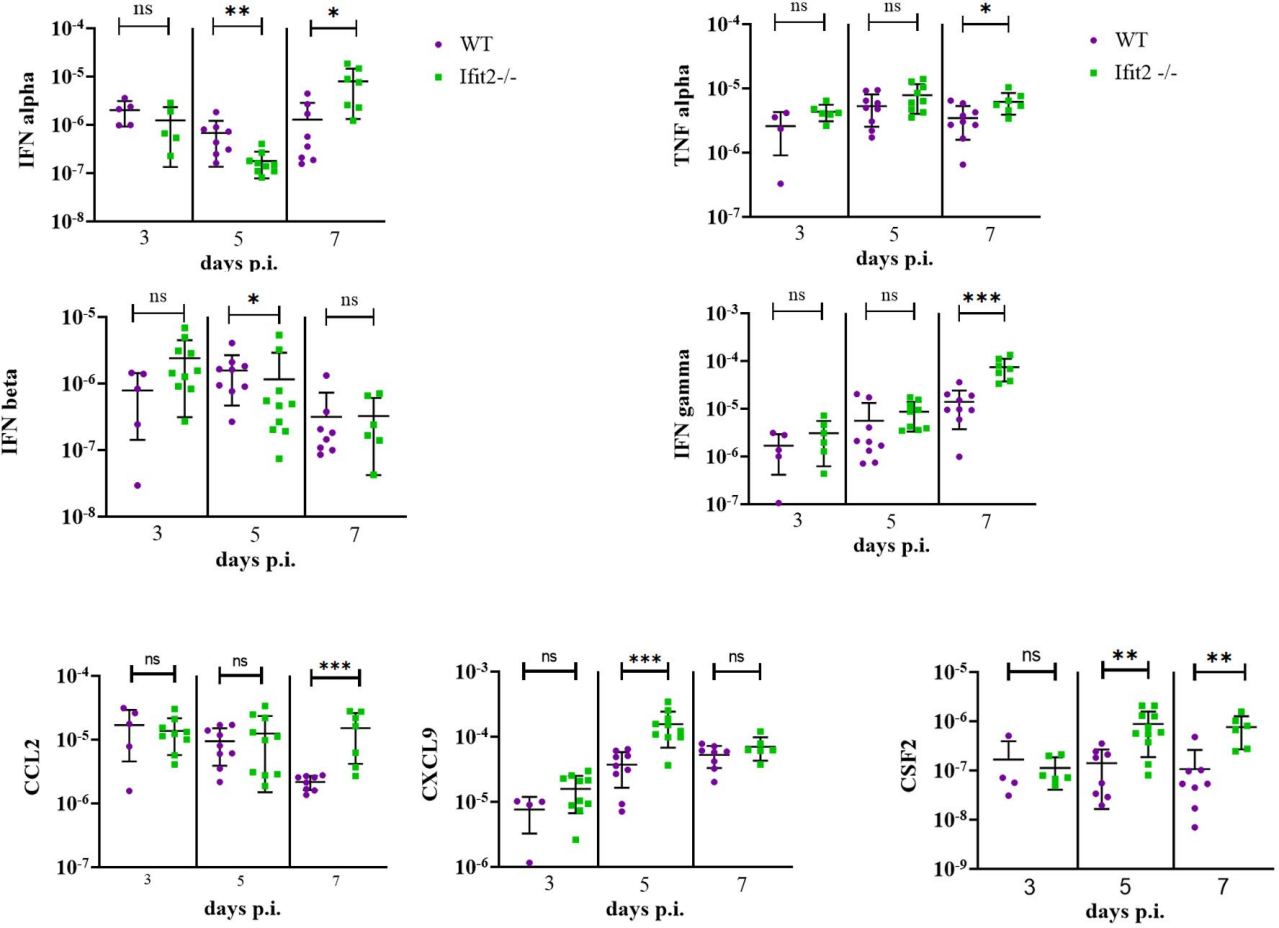
Differential mRNA expression levels of selected proinflammatory cytokines and chemokines in brains of infected Ifit2^-/-^ and WT mice. RNA extracted from individual brain tissues of RSA59 infected WT and Ifit2^-/-^ mice at day 3, 5, and 7 p.i. was analysed for mRNA levels of the indicated cytokines and chemokines by Real time PCR. Solid lines represent the mean levels of mRNA expression (n = 4–11). Asterix (*) represents differences that are statistically significant by Student’s unpaired t-test analysis (Mann-Whitney Test) (*P<0.05, **P<0.01, ***P<0.001).

Impaired cellular infiltration and microglia/macrophage activation, despite elevated viral loads in Ifit2^-/-^ compared to WT mice, suggested dysregulation of IFNα/β as well as proinflammatory mediators, as noted in previous studies [16]. Of note, young mice are more susceptible to numerous neurotropic infections possibly due to distinct patterns of homeostatic and inducible levels of factors in the IFNα/β pathway [39]. Assessment of IFNα mRNAs revealed no difference between the mouse groups at day 3 p.i., but reduced levels in Ifit2^-/-^ mice at day 5 p.i. (Fig 5), when viral load was significantly increased relative to WT mice. However, levels of IFNα mRNAs were increased at day 7 p.i., suggesting a delayed response. IFNβ expression peaked at day 3 p.i. in Ifit2^-/-^ mice and declined by day 5 and 7 p.i., but mRNA levels were only significantly lower compared to WT at day 5 p.i.

Several other mRNAs encoding proinflammatory cytokines affecting BBB integrity, antigen presentation, and antiviral functions, namely IFNℽ, IL6, TNF, CSF2 and IL1β, were also tested for differential expression (Fig 5). No significant differences were observed in any of these cytokines at day 3 p.i. While IFNγ, TNF and CSF2 mRNAs were all high in Ifit2^-/-^ relative to WT brains at day 5 or 7 p.i. IL6 and IL1β mRNA levels did not show any significant differences between the groups. RNA from brains of WT and Ifit2^-/-^ mice were also compared for mRNA expression levels of selected chemokines known to recruit neutrophils (CXCL1), monocytes and natural killer cells (CCL2, CCL5), and T lymphocytes (CXCL9, CXCL10 and CCL5[40] with varying selectivity. CCL2 mRNA was upregulated in Ifit2^-/-^ mice compared to WT mice at day 7 p.i. CXCL1, CXCL10 and CCL5 mRNA levels did not show significant variation between WT and Ifit2^-/-^ mice brain at any days (3–7) p.i. By contrast, CXCL9, a chemokine induced by IFNγ was only elevated at day 5 p.i. in Ifit2^-/-^ mice. Overall, elevated mRNAs expressing the selected chemokines in Ifit2^-/-^ relative to WT mice correlated with increased viral load. Nevertheless, relative to the log10-fold differences in viral load, the increase in chemokines appeared quite moderate (Fig 5).

### Ifit2 deficiency reduces CD45^hi^ leukocyte CNS infiltration affecting mainly lymphocytes

**Fig 6:**
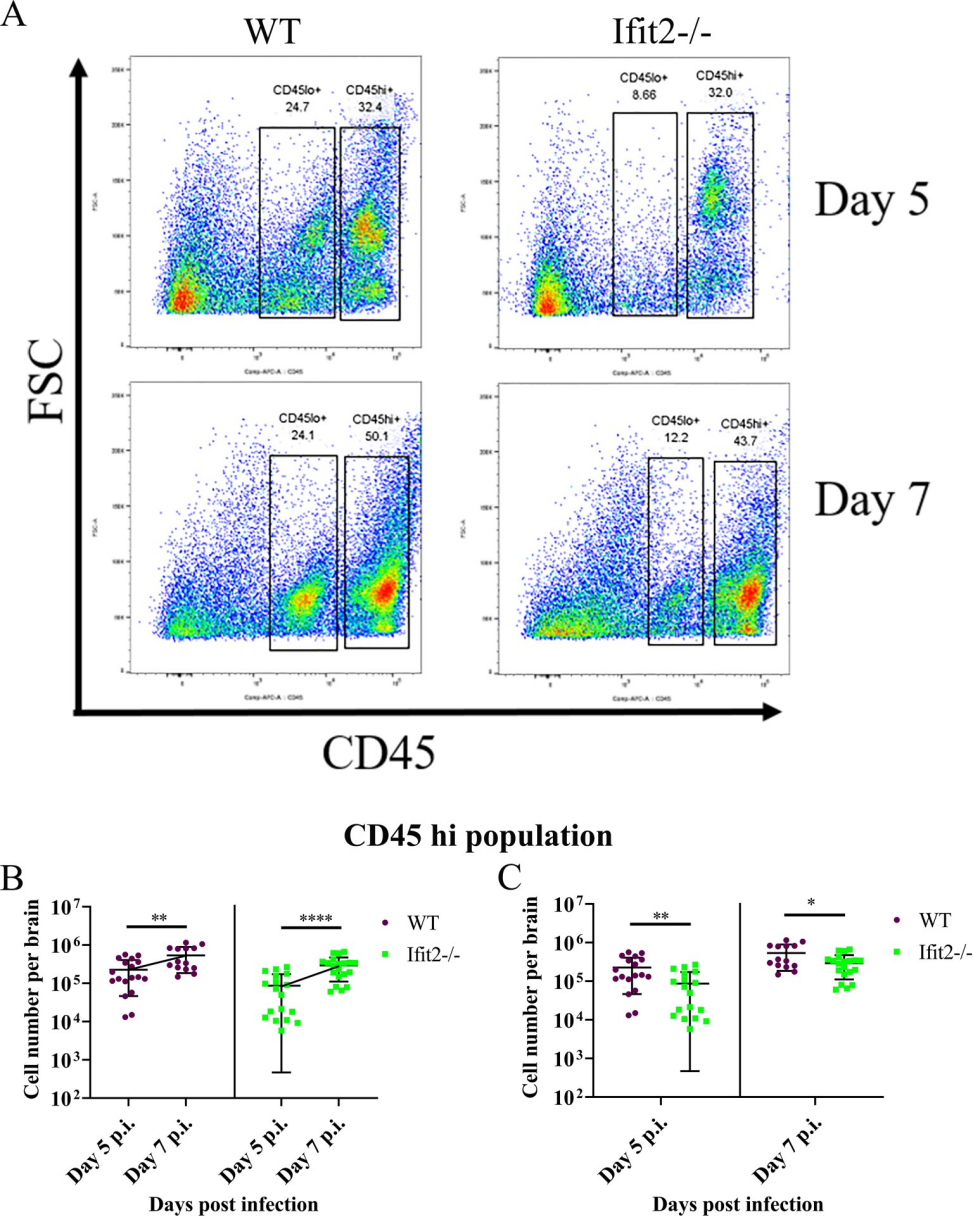
Ifit2 deficiency impairs CD45^+^ leukocyte migration into the CNS at day 5 and 7 post infection. Brains from WT and Ifit2^-/-^ mice infected with RSA59 were harvested at days 3, 5, and 7 p.i. for flowcytometric analysis. Purple colour denotes WT and green represents Ifit2^-/-^ mice. Panel A shows representative flow cytometry plots at day 5 and 7 p.i., indicating percentages of CD45^hi^and CD45^lo^ cells in brains after gating on live cells. Absolute numbers of CD45^hi^ cells are graphically represented across timepoints (Panel B) and in between WT and Ifit2^-/-^ groups for day 5 p.i., and day 7 p.i. (Panel C) to better represent respective statistics. Graphs represent n = 14–20 mice from 3 separate experiments. Asterix (*) represents differences that are statistically significant by Student’s unpaired t-test analysis. (*P<0.05, **P<0.01).

Activated CD45^hi^ leukocytes infiltrate into the RSA59 infected CNS, with the innate responders comprising neutrophils and monocytes followed by T cells [41]. Flow cytometric analysis of CNS cells was thus performed to assess differences in infiltration of specific leukocyte populations in RSA59 infected WT versus Ifit2^-/-^ mice at day 3, 5 and 7 p.i. Gating on total CD45^+^ cells allowed distinction between CD45^lo/in^ microglia and CD45^hi^peripheral infiltrating leukocytes (Fig 6A), while CD11b was used as a marker for myeloid cells including neutrophils and monocyte derived macrophages. A comparatively small population of CD45^hi^ cells was evident at day 3 p.i. in both WT and Ifit2^-/-^ infected mice (data not shown). The CD45^hi^ population increased markedly at day 5 and 7 p.i. (Fig 6B) in both WT and Ifit2^-/-^ infected mice (Fig 6B). However, CD45^hi^ cells were significantly lower in Ifit2^-/-^ infected mice compared to WT infected mice at both day 5 and 7 p.i. (Fig 6C).

**Fig 7:**
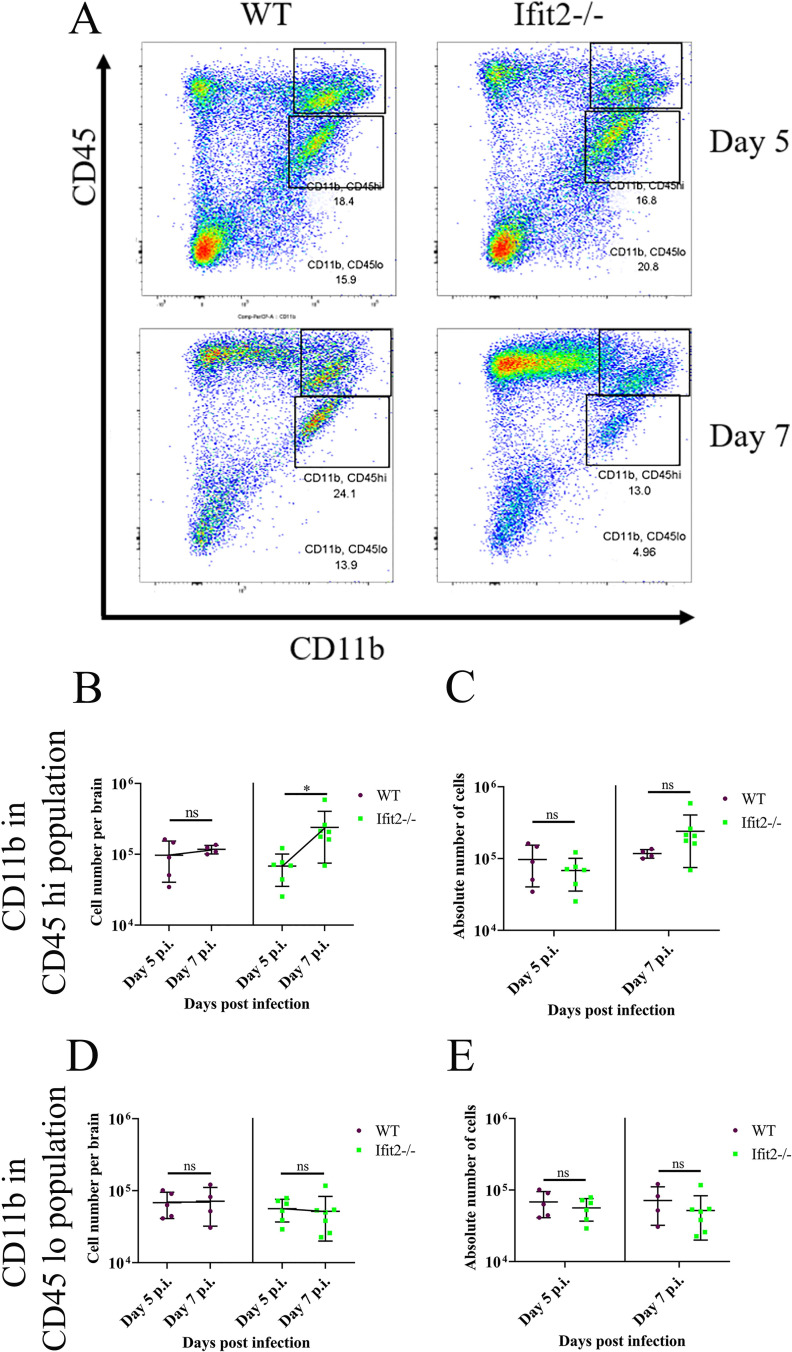
Ifit2 deficiency does not alter myeloid cell populations in the brain. Cell suspensions from brains of infected WT and Ifit2^-/-^ mice were analysed as described in Fig 6 by flow cytometry, following staining for CD45 and the myeloid marker CD11b. Panel A shows representative flow cytometry plots, indicating percentages of CD11b^+^CD45^hi^ and CD11b^+^CD45^lo^ cells at days 5 and day 7 p.i. Absolute numbers of CD11b^+^CD45^hi^ cells are graphically represented across timepoints (Panel B) and in between WT and Ifit2^-/-^ groups for day 5 p.i., and day 7 p.i. (Panel C) to better represent respective statistics. Similarly, absolute numbers of CD11b^+^CD45^lo^ cells are graphically represented for same group at each timepoint (Panel D) and in between WT ad Ifit2^-/-^ groups at two timepoints (Panel E). Graphs represent n = 4–7 mice and the experiment were repeated 2–3 times. Asterix (*) represents differences that are statistically significant by Student’s unpaired t-test analysis. (*P<0.05).

**Fig 8:**
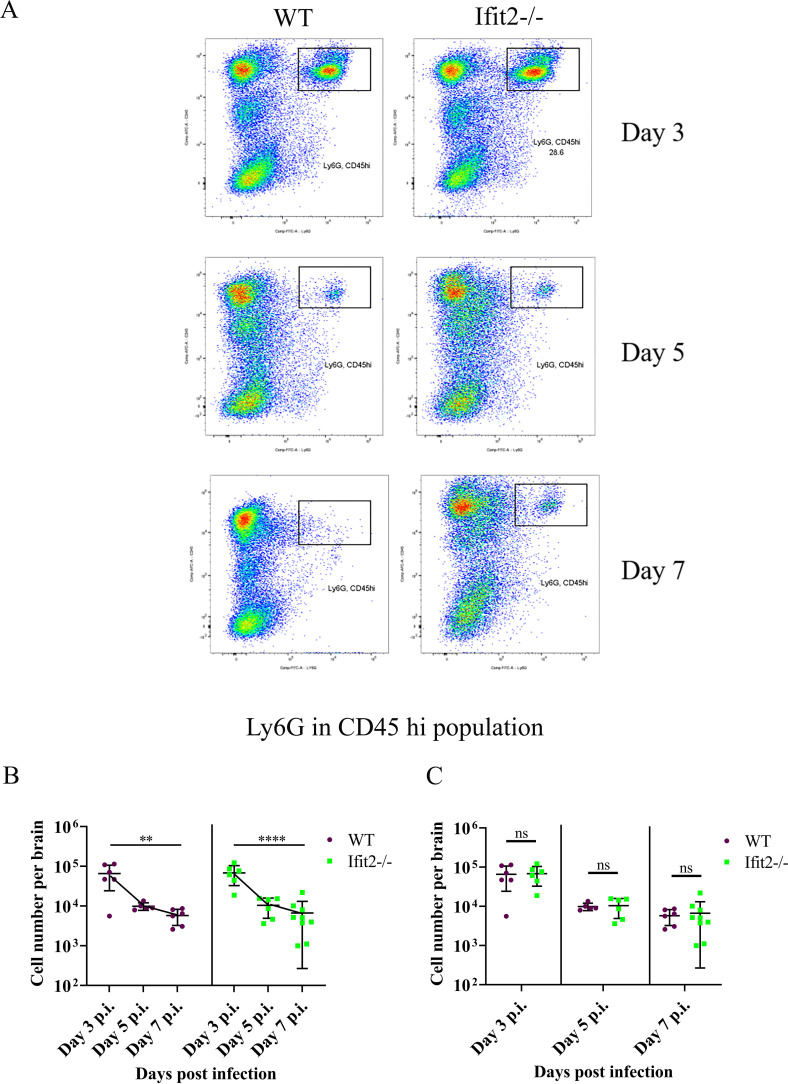
Ifit2 deficiency does not alter neutrophil migration into the CNS. Cell suspensions from brains of infected WT and Ifit2^-/-^ mice were analysed as described in Fig 6 by flow cytometry following staining for CD45 and the neutrophil marker Ly6G. Panel A shows representative flow cytometry plots at days 3,5,and 7 p.i. Absolute numbers of Ly6G^+^CD45^hi^ cells are graphically represented across timepoints for each group (Panel B) and between WT and Ifit2^-/-^ groups for day 3 p.i., 5 p.i., and day 7 p.i. (Panel C) to better represent respective statistics. The experiment is repeated twice and representative graphs are presented with n = 5–9. Asterix (*) represents differences that are statistically significant by Student’s unpaired t-test analysis. (**P<0.01, ****P<0.0001).

To address whether reduced leukocyte infiltration affected specific cell populations, we initially monitored early infiltrating CD45^hi^CD11b^+^ myeloid cells (Fig 7A). A significant increase in CD45^hi^CD11b^+^ cells was only noted between days 5 and 7 p.i. in Ifit2^-/-^ mice, but not in WT mice (Fig 7B), however CD45^hi^ CD11b^+^ cell numbers were not significantly different between the two groups (Fig 7C). There were also no significant differences in numbers of CD45^lo/int^ CD11b^+^ microglia across day 5 and 7 p.i., as well as comparing WT to Ifit2^-/-^ infected mice (Fig 7D and 7E). Staining for Ly6G to mark neutrophils (Fig 8A) revealed an early peak at day 3 p.i. with a subsequent decline in both groups, with no significant differences at any time point (Fig 8B and 8C). Reduced infiltration of CD45^hi^ leukocytes in Ifit2^-/-^ was thus not attributed to neutrophils or monocyte derived macrophages.

**Fig 9:**
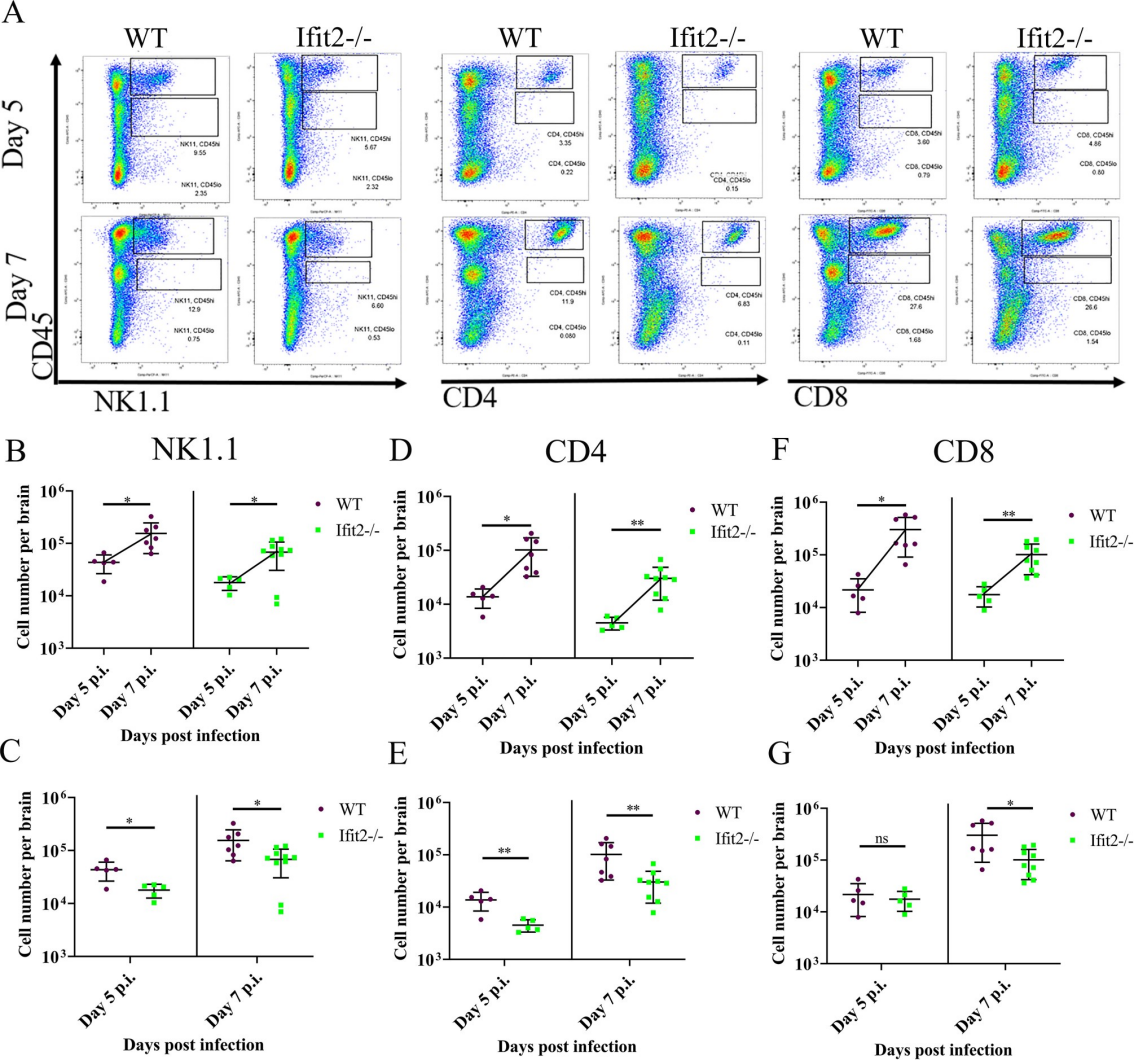
Ifit2 deficiency significantly impairs migration of NK1.1, CD4 and CD8 T cells among the CD45^hi^ population at acute stage of RSA59 infection. Dot Plot representation of the percentage of NK1.1, CD4 and CD8 expressing cells were gated from CD45^hi^ populations in the brain lysates, which was gated on live cells for both day 5 and 7 p.i. (Panel A). Purple colour denotes WT and green colour denotes Ifit2^-/-^ mice. Absolute number of NK1.1 expressing cells gated from CD45^hi^ population for both the WT and Ifit2^-/-^ total brain lysate is comparatively represented across timepoints (day 5 and day 7) (Panel B) and also between WT and Ifit2^-/-^ mice for day 5 p.i., and day 7 p.i. (Panel C). Absolute number of CD4 expressing cells gated from CD45^hi^ population for both the WT and Ifit2^-/-^ total brain lysate is comparatively represented across timepoints (day 5 and day 7) (Panel D) and also between WT and Ifit2^-/-^ mice for day 5 p.i., and day 7 p.i. (Panel E). Absolute number of CD8 expressing cells gated from CD45^hi^ population for both the WT and Ifit2^-/-^ total brain lysate is comparatively represented across timepoints (day 5 and day 7) (Panel F) and also between WT and Ifit2^-/-^ mice for day 5 p.i., and day 7 p.i. (Panel G). The data were pooled from two independent experiments with n = 5–10. Asterix (*) represents differences that are statistically significant by Student’s unpaired t-test analysis. (*P<0.05, **P<0.01).

Brain derived cells were also assessed for NK1.1^+^ NK cells, as well as CD4^+^ and CD8^+^ T cell subsets (Fig 9A). Both WT and Ifit2^-/-^ mice revealed a prominent increase in all three lymphocyte populations between days 5 and 7 p.i. (Fig 9B, 9D and 9F). By contrast, although CNS NK1.1^+^ and CD4^+^ T cells also increased between days 5 and 7 p.i. in Ifit2^-/-^ mice, their absolute numbers were significantly lower compared to WT mice at both day 5 and 7 p.i. (Fig 9C and Fig 9G, Fig 9E). CD8^+^ cells also increased in the CNS between days 5 and 7 p.i in both groups (Fig 9F). While their numbers were similar at day 5 p.i., they were lower at day 7 p.i. in the absence of Ifit2 (Fig 9G). The impaired accumulation of NK1.1+ and CD4+ T cells was more prominent compared to CD8+ T cells in Ifit2^-/-^ vs. WT infected mice. The results suggest that the smaller CD45^hi^ population in Ifit2^-/-^ relative to WT mice is primarily due to lower NK1.1^+^ and CD4^+^ T cell populations both at day 5 and 7 p.i.

### Ifit2^-/-^ infected mice showed restricted CX3CR1 expression in both microglia and infiltrating monocytes

**Fig 10:**
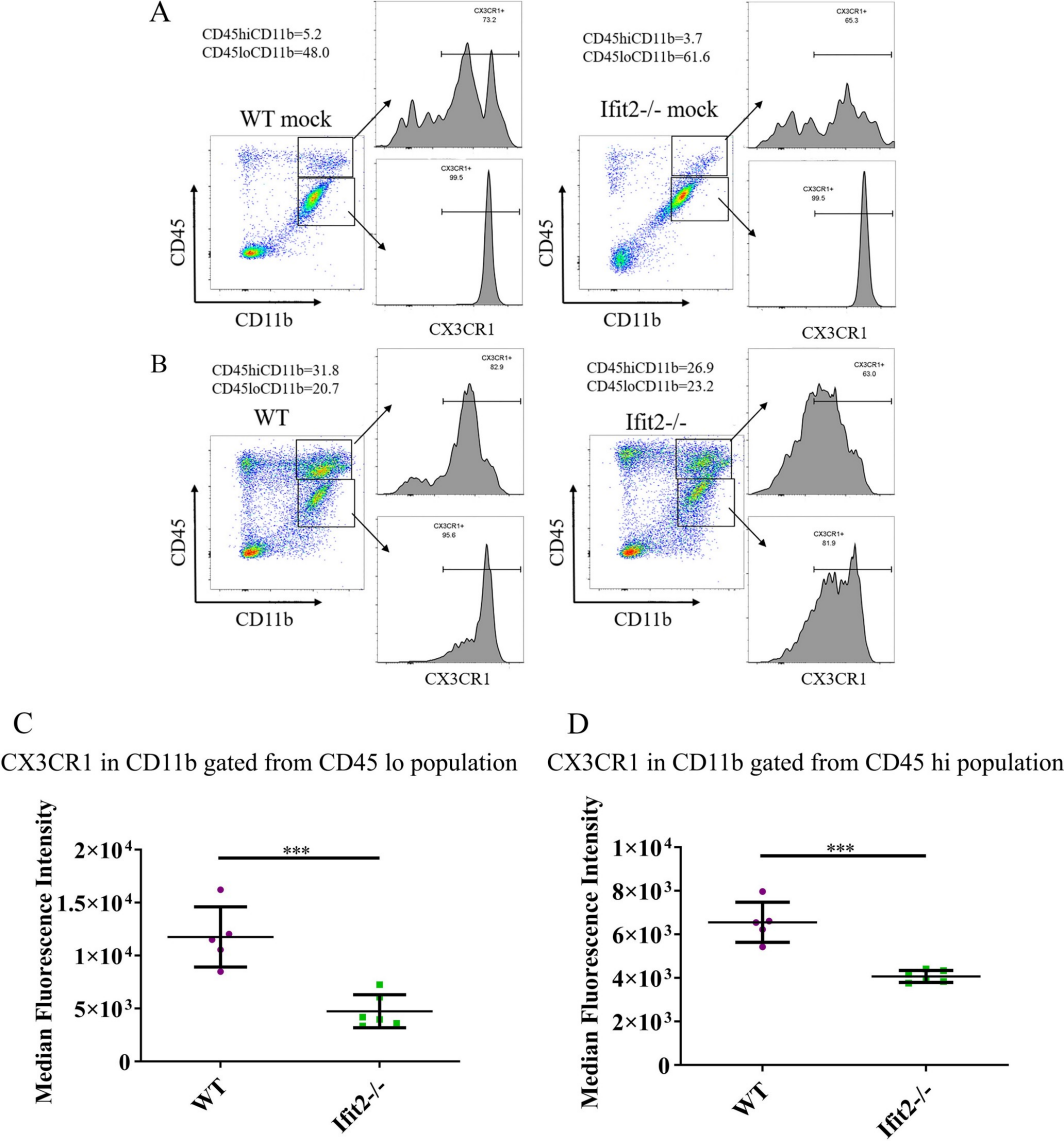
Ifit2 deficiency decreases CX3CR1 expression in microglia following infection. Brain derived cells from WT and Ifit2^-/-^ mice either mock infected (Panel A) or infected with RSA59 (Panel B) were stained for CD45, CD11b and CX3CR1 expression at 5 days p.i. Dot plots show gating and percentages of CD45^hi^ CD11b^+^ and CD45^lo^ CD11b^+^ populations as indicated. Histograms show CX3CR1 expression on the respective gated myeloid cell populations as indicated by arrows. Panels C and D show Median Fluorescent Intensity of CX3CR1 expression by CD45^lo^ CD11b^+^ microglia and CD45^hi^ CD11b^+^ infiltrates of WT (Purple) and Ifit2^-/-^ (Green) mice, respectively.The experiment is repeated 2–3 times with n = 5–6. Asterix (*) represents differences that are statistically significant by Student’s unpaired t-test analysis. (***P<0.001).

Flow data combined with immunohistochemistry revealed no difference in the number of CD11b^+^ microglia/monocytes, but the extent of microglial activation assessed by Iba-1 morphology was significantly reduced in Ifit2^-/-^ mice. Impaired microglial activation might be related to reduced lymphocyte recruitment, although no significant changes were observed in the CXC or CC chemokines as shown by their mRNA expression. Based on the emerging importance of the CX3CR1- CX3CL1 axis in microglia function as well as leukocyte adhesion and migration, we measured the level of CX3CR1 expression in brain derived microglia and leukocytes from mock and virus infected WT and Ifit2^-/-^ mice by flow cytometry (Fig 10). The median fluorescence intensity for CX3CR1 was measured from both CD45^lo^ and CD45^hi^ CD11b^+^ populations. CX3CR1 expression was high on the CD45^lo^CD11b^+^ microglia population in both mock infected groups, consistent with abundant CX3CR1 on CNS resident naïve microglia (Fig 10A). Importantly, there were no significant differences between mock infected WT and Ifit2^-/-^ mice (Fig 10A). CX3CR1 expression was also evident in brain derived CD45^hi^ CD11b^+^ myeloid cells in mock infected mice, although the expression pattern differed (Fig 10A). Whereas there was a clear split into CX3CR1^hi^, CX3CR1^int^ and CX3CR1^-^ cells in WT brains, the CX3CR1^hi^ population in Ifit2^-/-^ brains expressed reduced CX3CR1 levels (Fig 10A). Infection only modestly reduced CX3CR1 in WT CD45^lo^CD11b^+^ microglia but the overall peak MFI was not altered (Fig 10B). By contrast, Ifit2^-/-^ microglia from infected mice exhibited a significant loss in CX3CR1 expression at day 5 p.i. as revealed by the large downward shift in MFI (Fig 10B). The CD45^hi^ CD11b^+^ myeloid cell population from infected WT mice showed an overall loss of the CX3CR1^hi^ population, although the vast majority retained CX3CR1 expression (Fig 10D). Similarly, CD45^hi^CD11b^+^ cells from infected Ifit2^-/-^ mice exhibited overall lower MFI of CX3CR1 on Ifit2^-/-^ compared to WT myeloid cell infiltrates (Fig 10D). Similarly, CD45^lo^ CD11b^+^ cells from infected Ifit2^-/-^ mice expressed overall lower CX3CR1, resulting in overall lower MFI of CX3CR1 on Ifit2^-/-^ compared to WT counterparts (Fig 10C). These results suggest that Ifit2 deficiency results in a significant loss of CX3CR1 expression on microglia, and to a lesser extent on myeloid cells in response to infection.

**Fig 11:**
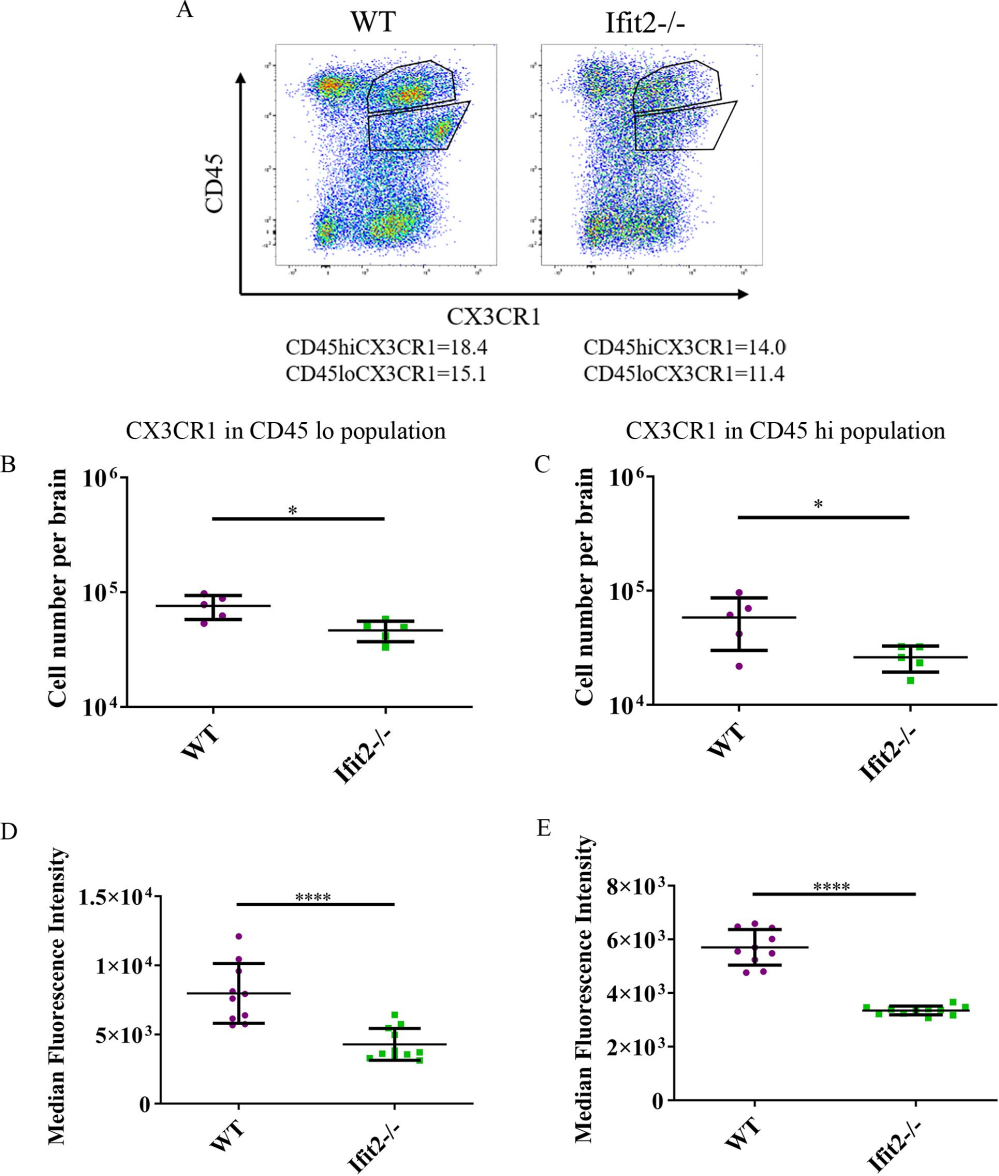
Ifit2 deficiency resulted in downregulation of CX3CR1 upon RSA59 infection. Brain derived cells from WT and Ifit2^-/-^ mice infected with RSA59 were stained for CD45 and CX3CR1 expression at 5 days p.i. Dot plots show gating and percentages of CD45^hi^ and CD45^lo^ populations as indicated. Dot Plot representation of the percentage of CX3CR1 expressing cells were gated from CD45^hi^ and CD45^lo^ populations in brain lysates at day 5 p.i. (Panel A; gated on live cells). Purple colour denotes WT and green colour denotes Ifit2^-/-^ mice. Panels B and C show absolute numbers of CX3CR1 expressing CD45^lo^ cells and CD45^hi^ cells as gated in Panel A from WT and Ifit2^-/-^ mice, respectively. Panels D and E show Median Fluorescent Intensity of CX3CR1 expressing CD45^lo^ and CD45^hi^ cells, respectively. The experiment is repeated 2–3 times with n = 5 for absolute number of cells and n = 10–11 for median fluorescent intensity. Asterix (*) represents differences that are statistically significant by Student’s unpaired t-test analysis. (*P<0.05, ****P<0.0001).

We also compared CX3CR1 expression in total CD45^lo^ and CD45^hi^ populations in both groups of infected mice at day 5 p.i. (Fig 11). Consistent with results above, CX3CR1 expression was significantly lower in both CD45^lo^ and CD45^hi^ populations (Fig 11A–11C). In addition to reduced absolute numbers of cells, CX3CR1 median fluorescent intensities in CX3CR1^+^ populations were significantly reduced in both CD45^hi^ and CD45^lo^ cells of Ifit2^-/-^ compared to WT counterparts (Fig 11D and 11E).

As CX3CR1 is known to be expressed in different subpopulations of T-lymphocytes, we calculated the expression level of CX3CR1 in individual subpopulations of T cells (NKT cells, CD4^+^ T cells and CD8^+^ T cells). No significant changes in the median fluorescent intensity of CX3CR1 expression were observed in NK+ T cells, CD4+T cells and CD8+ T cells between Ifit2^-/-^ infected mice and WT infected mice (data not shown). In contrast to distinct regulation of CX3CR1, analysis of the ligand revealed no significant changes based on fractalkine mRNA expression levels between infected WT and Ifit2^-/-^ mice at either day 3 or day 5 p.i. (data not shown).

In summary, Ifit2 plays a role in maintaining CX3CR1 surface expression in both CD45^hi^ CD11b+ monocyte derived macrophages and CD45^lo/int^ CD11b+ CNS resident microglia following MHV-RSA59 infection. Reduced levels of CX3CR1 in Ifit2^-/-^ relative to WT inflamed brains may be responsible for the impairment of morphological microglial activation and lymphocyte migration into the CNS.

## Discussion

Ifit2 is only expressed at low levels in most cell types under non-pathogenic conditions except for a few myeloid cells, but shows rapid and significant induction upon viral infection [1,11,13, 14,16]. Ifit2 contributes significantly towards enhancing both antiviral cell-intrinsic and cell-extrinsic host immune responses to limit the virus spread to neighboring cells. Several studies of neurotropic virus infections have shown that Ifit2 deficiency results in higher disease severity and mortality [13–16]. Although Ifit2 mediated protection may partially involve IRF3 mediated promotion of IFNα/β production[16], other cellular mechanisms may further be involved. The study herein reveals a novel putative role of Ifit2 in microglial activation and their sustained surface expression of CX3CR1, a chemokine receptor known to be involved in neuronal communication [42] as well as leukocyte infiltration through BBB and successive viral clearance.

Enhanced disease severity of RSA59 infected Ifit2^-/-^ relative to WT mice was accompanied by increased viral load and spread throughout the brain, although development of encephalitis marked by perivascular cuffing and formation of microglial nodules was greatly impaired. Furthermore, despite their vast viral load, Ifit2^-/-^ mice did not show a corresponding significant increase in mRNA expression of cytokines and chemokines compared with WT mice. Mock infected Ifit2^-/-^ and WT mice did not exhibit any observable histological differences in the absence of Ifit2 suggesting virus induced alterations in inflammation.

A reduction in CNS infiltrating CD45^hi^ cells in Ifit2^-/-^ mice compared to WT mice was largely associated with impaired recruitment of NK-T^+^, CD4^+^ and CD8^+^ lymphocytes, but not altered neutrophils/macrophages. To assess a mechanism linking impaired virus clearance with impaired microglial activation and reduced lymphocyte infiltration we evaluated a possible role for dysregulated chemokine/chemokine receptor interactions involved in neuronal-microglia crosstalk. Chemokine-chemokine receptor [43–45] interactions transduce signals that result in cytoskeletal reorganization, integrin activation, and other functions, leading to increased adhesion and migration of leukocytes [46,47]. Specifically the interaction between microglial CX3CR1 and CX3CL1 expressed by neurons regulates microglial activation and cross talk between neurons and glia in several neurological diseases [25,48,49] like Tau pathology [50], Alzheimer’s Diseases (AD) [51], Multiple Sclerosis (MS)[52], Amyotrophic Lateral Sclerosis (ALS)[53,54], and HIV-induced encephalitis [55]. In addition, CX3CR1 on leukocytes is known to interact with endothelial CX3CL1 to promote arrest, recruitment and migration of peripheral monocytes/lymphocytes through the BBB [19,28,51,54,56]. The CX3CR1-CX3CL1 axis also plays a major role in vascular inflammation [57]. Our study showed reduced expression of CX3CR1 on microglia and reduced infiltrating leukocytes in infected Ifit2^-/-^ compared to WT mice. However, analysis of expression levels in mock infected mice revealed that CX3CR1 expression was similarly high in both groups, suggesting CX3CR1 was more selectively downregulated in the absence of Ifit2 in both microglia and infiltrating myeloid cells. This finding may be associated with both impaired morphological microglial activation and lower peripheral lymphocyte recruitment in Ifit2^-/-^ mice. The linkage between Ifit2 deficiency and the CX3CR1/CX3CL1 pathway can be indirect due to increased viral replication and potential activation of pattern associated factors resulting in CX3CR1 downregulation, reminiscent of exposure to LPS [58] or distinct signals provided by surface bound or shed CX3CL1 [58]. Alternatively, the lack of Ifit2 may attenuate translational efficiency of a subset of cellular mRNAs involved in the CX3CR1- CX3CL1 axis, a mechanism recently revealed during influenza virus infection [59]. Despite numerous studies comparing WT, CX3CR1-/-, and CX3CR1-/+ mice, there is surprisingly little information on regulation of CX3CR1 or CX3CL1 expression at the transcriptional, translational or posttranslational levels [60]. The CX3CR1 signaling cascade can activate several transcription factors (NfkB,CREB, STAT, AP-1) thereby potentially activating a wide range of functions such as transcription regulation of cytokine, cytoskeletal rearrangement, migration, apoptosis, and proliferation [25,61]. Furthermore, the CX3CL1 ligand is synthesized as a membrane bound surface chemokine, which can be processed to release the soluble form by activated membrane proteases [25,49]. The checkpoint function of CX3CR1 signaling in limiting microglial activation, and additional mediators of microglial activation during microbe-induced neuroinflammation will necessitate conditional and cell type specific manipulation of both Ifit2 and CX3CR1 to dissect their crossregulation.

Further, lymphocyte recruitment is regulated both at the vasculature as well as within the parenchyma [62–64]. We can also not rule out an indirect effect of Ifit2 on activation of virus specific lymphocytes, yet no deficits in IFNγ mRNA levels in the brains indicate NK and T cells are functional. Our study, thus, for the first time suggests that Ifit2 plays a major role in inducing microglial activation either directly or indirectly by regulating CX3CR1 expression to mount the host immunity. Current data thus provide an ideal platform to investigate an alternative cellular pathway of rolling leukocyte adhesion and migration through the BBB by means of fractalkine expressed on inflamed endothelium acting as a vascular gateway for myeloid effector cells (CX3CR1-expressing cells) by rapidly capturing them from the blood and promoting migration into the tissue. Fractalkine may enhance extravasation of leukocytes by mediating cellular adhesion through the initial tethering and transmigration steps [28,29,65]. This model is thus suitable to understand integrin biology in correlation with cellular architecture of the BBB. Nonetheless, the CX3CL1-CX3CR1 axis also adds a novel angle to the role of Ifit2 in the microglial activation process, and further studies warrant exploring the role of Ifit2 in neuro-microglial communication.

It will also be informative to assess the kinetics of the disease severity at a lower dose of the virus and examine the timely infiltration of T lymphocytes into the CNS. The migration of peripheral T cells is of significant importance in mounting host immunity against RSA59 infection and its associated disease severity, while their interaction with microglia is important to restore homeostasis [41]. A large number of studies attempt to understand the dynamic migration of T cells and their associated pathogenic or protective functions. Taking insights from the studies in Ifit2^-/-^ mice at the acute stage, we can suggest that RSA59 infection in Ifit2^-/-^ mice can be an appropriate model to study the nexus between T cells and microglia and their combined effect on host immunity and antiviral immune responses.

In summary, the current study implies an important role of Ifit2 in CNS resident microglia activation and associated lymphocyte migration to mount host immunity. We also identified for the first time that Ifit2 deficiency impairs CX3CR1 expression. While our study employs an experimental mouse system to study the role of Ifit2 in neuroinflammation and antiviral immune responses, the insights will have a long-term impact in understanding human neurodegenerative diseases, especially MS. Genome- wide association studies have already started to identify the association of Ifit2 in the disease pathogenesis of autoimmune Systemic lupus erythematosus (SLE) [66], Multiple Sclerosis (MS) [67], Amyotrophic Lateral Sclerosis (ALS), Alzheimer (AD) and Parkinson’s disease (PD)[68] and providing new insights into the specifications of ISG actions. The limitation, however, lies in the absence of a humanized mouse model for Ifit2.

While it has been widely accepted that upregulation of Ifit2 is protective upon several virus infections like Rabies Virus [11], lethal VSV [12,13], WNV [14], and Sendai virus (SeV)[15] infection, the exact regulatory role is not defined yet. Moreover, among the coronaviruses, MHV is the only model used to understand the role of Ifit2. Given the importance of Ifit2 in providing host immunity, it is the need of an hour to investigate its regulatory role in the lethal HCoV SARS-CoV2 infection which has induced COVID-19 pandemic worldwide and is keeping the entire world in real tenterhooks [69]. This virus belongs to the same family of β-coronaviruses as MHV. Similar to the MHV-RSA59 model, COVID-19 patient serum samples tested low for both IFNβ and the IFNγ family of interferons at the acute phase of inflammation [70–72]. Although the IFN response was limited, a robust ISGs upregulation, including interferon-induced transmembrane protein (IFITMs’) response was observed. A report showed that a single-nucleotide polymorphism rs12252 in IFITM3 gene was correlated with higher disease severity in COVID-19 [73]. Moreover, previous studies had also suggested IFITM limiting SARS-CoV replication and invasion (mediated by the spike protein), independently of the expression of viral receptors [74]. The protective role of Ifit2 has also been observed in Sendai virus lung pathogenesis [15].

Although a dysregulated homeostasis in the lungs has proven to be fatal in COVID-19, it should not be ignored that the inability to procure oxygen by the lungs, might be due to defects in the respiratory control centre of the brainstem. The neuro-invasive potential for prototype HCoV strains like OC43 and 229E in the human brains have been documented previously [75,76]. SARS-CoV particles were also found in the brains of infected patients [77]. SARS-CoV2 has also been observed to be present in the cerebrospinal fluid by genome sequencing in one of the studies[78]. Several pieces of evidence suggested mild to severe neurological manifestations in COVID-19 patients [70,79,80]. As Ifit2 plays a major role in limiting neurotropic RSA59 viral replication by regulating microglial activation in the CNS, it will be critical to dissect the role of Ifit2 in peripheral as well as CNS host immunity against HCoVs.

## Materials and methods

### Ethics statement

All studies were carried out in strict accordance with all provisions of the Animal Welfare Act, the Guide for the Care and Use of Laboratory Animals, and the PHS Policy on Humane Care and Use of Laboratory Animals. All animal experiments were performed in compliance with protocols approved by the Cleveland Clinic Institutional Animal Care and Use Committee (PHS assurance number A3047-01).

### Virus infection in mice

C57BL/6 mice and Homozygous Ifit2^-/-^ mice on the C57BL/6 background were obtained from the breeding colony of LRI Biological Resources Unit, Lerner Research Institute, Cleveland Clinic, USA as previously described.

All mice were housed under pathogen-free conditions at an accredited facility at the Cleveland Clinic Lerner Research Institute and used at 4 to 5 weeks of age. The hepatotropic and neurotropic recombinant, EGFP expressing strain of MHV-A59 known as RSA59 was used in the study. The virus was propagated in 17Cl1 cells and plaque assayed on DBT astrocytoma cell monolayers. Mice were infected intracranially in the right hemisphere with 2,000 PFUs of RSA59 diluted in endotoxin free, filter-sterilized PBS-BSA (Dulbecco’s phosphate-buffered saline +0.75% BSA) in a final volume of 20 μl. Age-matched mice were mock infected with PBS-BSA or not infected and kept as non-infected control. Clinical disease severity was graded daily using the following scale: 0, no disease symptoms; 1, ruffled fur; 1.5, hunched back with mild ataxia: 2, Ataxia, balance problem and hind limb weakness: 2.5 one leg completely paralyzed, motility issue but still able to move around with difficulties; 3, severe hunching/wasting/both hind limb paralysis and mobility is severely compromised; 3.5 Severe distress, complete paralysis and moribund 4, dead. Our study also investigated for any phenotypic or pathological symptoms between age matched control (non-infected) and mock infected WT and Ifit2^-/-^male mice, but no such significant gross phenotypic clinical symptoms or histological changes were observed.

### Viral titer estimation

Virus titers in the CNS were determined as described previously. Briefly, brains were homogenized individually in Dulbecco’s PBS using Tenbroeck tissue homogenizers. Homogenates were clarified by centrifugation at 400 g for 7 min at 4°C, and supernatants were stored at -80°C until thawed for plaque assay on DBT astrocytoma cell monolayers as described previously[16,30].

### Viral antigen spread in frozen sections

RSA59 infected WT and Ifit2^-/-^ mice (day 5 p.i.) were perfused transcardially with PBS, followed by cold PBS containing 4% PFA. Brains were harvested in 4% PFA for 6 h and then placed at 4°C for 4 h in 10% sucrose, followed by 30% sucrose overnight. Tissues were embedded with OCT medium (Tissue Tek, Hatfield, PA), sectioned sagitally with the help of a Cryotome (Thermo Scientific) to 5-10-μm thickness, and mounted on charged glass slides. Frozen tissue sections were washed with PBS at RT to remove cryomatrix. Tissues then were incubated for 1 h at RT with 1 M glycine in PBS to reduce nonspecific cross-linking, followed by a 10-min incubation at RT with 1 mg/ml NaBH4 in PBS to reduce autofluorescence. The slides were then washed with PBS and scanned for EGFP fluorescence. A Leica Aperio AT2 slide scanner (Leica Microsystems, GmbH, Wentzler, Germany) was used to scan images of whole slides at 20x magnification. High resolution images were acquired using a Leica SP8 confocal microscope (Leica Microsystems, GmbH, Wetzlar, Germany) that was purchased with funding from National Institutes of Health SIG grant 1S10OD019972-01.

### RNA extraction, reverse transcription and real time PCR analysis

RNA was extracted using TRIzol reagent (Invitrogen, Carlsbad, CA) according to the manufacturer’s instructions and subjected to real-time PCR analysis as described previously. Tissues were homogenized in TRIzol, treated with chloroform, and RNA was precipitated with isopropyl alcohol, washed with 75% ethanol, and resuspended in RNase-free water (Gibco/Invitrogen, Grand Island, NY). Following treatment with DNase I using a DNA Free kit (Ambion, Austin, TX) for 30 min at 37°C following the manufacturer’s instructions, 1μg RNA was converted to cDNA using the Moloney murine leukemia virus reverse transcriptase (Invitrogen) in buffer containing 25 mM deoxynucleoside triphosphate mix, 250 ng random hexamer primers and oligo(dT) (1:1 ratio) (Invitrogen). cDNA samples were diluted 10-fold in RNase-free water before analysis by quantitative real-time PCR using SYBR green master mix (Applied Biosystems, Foster City, CA) (36). The list of primers is attached in Table 1. All samples were run in quadruplicates on a 96-well plate using a 7500 fast real-time PCR system (Applied Biosystems) with an automatically set baseline and a manually set critical threshold (CT) at which the fluorescent signal becomes higher than the signals for all of the PCR pairs. Dissociation curves were used to confirm amplification of a single product for each primer pair per sample.

**Table 1:** List of Primers.

CCL2	5'-CATCCACGTGTTGGCTCA 3'-GATCATCTTGCTGGTGAATGAGT
Csf2	5'-GCATGTAGAGGCCATCAAAGA 3'-CGGGTCTGCACACATGTTA
Cxcl9	5'-CTTTTCCTCTTGGGCATCAT 3'-GCATCGTGCATTCCTTATCA
*Ifn α*	5'-CTTCCACAGGATCACTGTGTACCT 3'-TTCTGCTCTGACCACCTCCC
*Ifn β*	5'-CTGGCTTCCATCATGAACAA 3'-AGAGGGCTGTGGTGGAGAA
*Ifn ℽ*	5'-ATCTGGAGGAACTGGCAAAA 3'-TTCAAGACTTCAAAGAGTCTGAGG
*Tnf α*	5'-GGTCTGGGCCATAGAACTGATG 3'-AGCAGGTGTCCCAAAGAA
18S	5'-ATTGACGGAAGGGCACCACCAG 3'-CAAATCGCTCCACCAACTAAGAACG

### Histopathological and immunohistochemical analysis

#### Hematoxylin/eosin staining

Mock and RSA59 infected mice from both C57BL/6 WT and Ifit2^-/-^ groups were sacrificed at the peak of inflammation (day 5p.i.), and were perfused transcardially with PBS followed by PBS containing 4% paraformaldehyde (PFA). Brain, and spinal cord tissues were collected, post-fixed in 4% PFA overnight and embedded in paraffin. Brain and spinal cord tissues were sectioned at 5 μm and stained with hematoxylin/eosin (H&E) for evaluation of inflammation. Experiments were repeated four times with 4–5 mice in each group.

#### Immunohistochemical staining and quantification

Serial sections from brain and spinal cord were stained by the avidin–biotin–immunoperoxidase technique (Vector Laboratories) using 3, 3′-diaminobenzidine as substrate, and anti-Iba1 (Wako, 1:250), anti-GFAP (Sigma, 1:500), or Anti-N (1:50) as primary antibodies. Control slides from mock-infected or uninfected mice were incubated in parallel. 7–8 sections from each infected group were randomly selected from three different sets of experiments and the expression of viral antigen, GFAP and Iba1 was quantified. Briefly, whole slides were scanned in a Leica Aperio AT2 slide scanner (Leica Microsystems, GmbH, Wetzlar, Germany) at 20x magnification and analyzed in Aperio Imagescope version 10.0.36.1805 software (Aperio) and quantified. For quantification, brightfield images of whole brain sections were analyzed using open source software QuPath[81]. Whole tissue sections were selected as regions of interest for analysis, areas with tissue folding, damage or out of focus tissue were then excluded by manual annotation. Stain colors were separated into respective components by RGB color vector dependent color deconvolution. Using thresholding on the deconvolved stained images, positive pixel area was then measured as percentage.

### Flow cytometry analysis

Mice were perfused with PBS, and brains were homogenized in 4 ml of Dulbecco’s PBS (pH 7.4) using Tenbroeck tissue homogenizers. Following centrifugation at 450g for 10 min, cell pellets were resuspended in RPMI containing 25 mM HEPES (pH 7.2), adjusted to 30% Percoll (Sigma) and underlaid with 1 ml of 70% Percoll. Following centrifugation at 800 g for 30 minutes at 4°C, cells were recovered from the 30%-70% interface, washed with RPMI, and suspended in FACS buffer (0.5% bovine serum albumin in Dulbecco’s PBS). Fc receptors were blocked with 1% polyclonal mouse serum and 1% rat anti-mouse CD16/ CD32 (clone 2.4G2; BD Biosciences, San Jose, CA) monoclonal antibody (MAb) for 20 minutes. Specific cell types were identified by staining with fluorescein isothiocyanate (FITC)-, phycoerythrin (PE)-, peridinin chlorophyll protein (PerCP)-, or allophycocyanin (APC)-conjugated MAb for 30 minutes on ice in FACS buffer. Expression of surface markers was characterized with MAb (all from BD Biosciences except where otherwise indicated) specific for CD45 (clone Ly-5), CD4 (clone GK1.5), CD8 (clone 53–6.7), CD11b (clone M1/70), Ly-6G (clone 1A8), and NK1.1 (clone PK136). Samples were analyzed using a BD LSRFortesa flow cytometer (BD Biosciences) and FlowJo 10 software (Treestar, Inc., Ashland, OR).

### Statistical analysis

Clinical scores were analyzed by a log-rank (Mantel-Cox) test. Real-time PCR data *in vivo* were analyzed by an unpaired t-test (Mann-Whitney Test). All immunohistochemical analysis and flow cytometry analysis was performed by Student’s unpaired t-test. Data were analyzed using Prism software (GraphPad Prism5). Two-Way ANOVA analysis for clinical score and unpaired t-test was performed for viral titer analysis. (**P<0.01, ***P<0.001, ****P<0.0001).
